# Lateral Entorhinal Cortex Lesions Impair Local Spatial Frameworks

**DOI:** 10.3389/fnsys.2017.00030

**Published:** 2017-05-17

**Authors:** Maneesh V. Kuruvilla, James A. Ainge

**Affiliations:** School of Psychology and Neuroscience, University of St AndrewsSt Andrews, UK

**Keywords:** hippocampus, spatial memory, navigation, medial entorhinal cortex, object recognition, allocentric

## Abstract

A prominent theory in the neurobiology of memory processing is that episodic memory is supported by contextually gated spatial representations in the hippocampus formed by combining spatial information from medial entorhinal cortex (MEC) with non-spatial information from lateral entorhinal cortex (LEC). However, there is a growing body of evidence from lesion and single-unit recording studies in rodents suggesting that LEC might have a role in encoding space, particularly the current and previous locations of objects within the local environment. Landmarks, both local and global, have been shown to control the spatial representations hypothesized to underlie cognitive maps. Consequently, it has recently been suggested that information processing within this network might be organized with reference to spatial scale with LEC and MEC providing information about local and global spatial frameworks respectively. In the present study, we trained animals to search for food using either a local or global spatial framework. Animals were re-tested on both tasks after receiving excitotoxic lesions of either the MEC or LEC. LEC lesioned animals were impaired in their ability to learn a local spatial framework task. LEC lesioned animals were also impaired on an object recognition (OR) task involving multiple local features but unimpaired at recognizing a single familiar object. Together, this suggests that LEC is involved in associating features of the local environment. However, neither LEC nor MEC lesions impaired performance on the global spatial framework task.

## Introduction

The medial temporal lobe network centered on the hippocampus and entorhinal cortex has been shown to have a clear role in memory and navigation (O’Keefe and Nadel, 1978; Morris et al., 1982; Vargha-Khadem et al., 1997). One key feature of this network is that the medial (MEC) and lateral (LEC) entorhinal cortices provide distinct anatomical inputs into hippocampus (Insausti et al., 1997; Kerr et al., 2007; Canto et al., 2008; van Strien et al., 2009). This suggests that the principle role of the hippocampus is in integrating information received from each entorhinal sub-region (Naber et al., 2001; Witter, 2007; Morrissey and Takehara-Nishiuchi, 2014; Masurkar et al., 2017). The predominant theory of how information is processed in the hippocampal network is that spatial information from the MEC (Fyhn et al., 2004; Hafting et al., 2005; Barry et al., 2006; Sargolini et al., 2006; Savelli et al., 2008; Solstad et al., 2008; Lever et al., 2009; Kropff et al., 2015) is combined with non-spatial or item based information from the LEC (Hargreaves et al., 2005; Knierim et al., 2006; Deshmukh and Knierim, 2011; Deshmukh et al., 2012; Stouffer and Klein, 2013) to form item-in-spatial-context representations that underlie episodic memory (Hayman and Jeffery, 2008; Hasselmo, 2009; Eichenbaum et al., 2012). While the majority of this research has been carried out in rodents, such a functional dissociation between MEC and LEC is also consistent with evidence from human imaging studies (Schultz et al., 2012; Reagh and Yassa, 2014) and has been used to support models such as the binding item in context model (Ranganath, 2010).

These models have been very influential but in recent years there have been other attempts to describe how information is integrated within the hippocampal-entorhinal network to support navigation and memory. Following on from O’Keefe and Nadel’s influential theory, it has been proposed that the cognitive map within the hippocampus is supported by spatially selective information from both the MEC and LEC (Knierim et al., 2013). The role of the MEC in encoding spatial information is well documented with a number of different types of spatial representations within MEC having been described. These include grid cells, head direction cells, border/boundary vector cells and speed cells (Hafting et al., 2005; Barry et al., 2006; Savelli et al., 2008; Solstad et al., 2008; Lever et al., 2009; Kropff et al., 2015). There is now growing support from lesion and single unit recording studies for the suggestion that LEC is involved in processing past and present locations of objects (Deshmukh and Knierim, 2011; Beer et al., 2013; Hunsaker et al., 2013; Tsao et al., 2013; Van Cauter et al., 2013; Wilson et al., 2013a,b; Chao et al., 2016; Keene et al., 2016). Our own work has shown that LEC is needed to associate together features of a local environment including spatial locations of objects (Wilson et al., 2013a,b). We see increased activity (measured using *c-fos* expression) in LEC when rats discriminate between novel and familiar combinations of features and rats with lesions of LEC perform at chance levels in tests of associative recognition memory while showing normal memory for individual features of an environment. These studies start to reconcile the roles of the hippocampal-entorhinal network in episodic memory and the cognitive map by describing how different types of information from our everyday experience are integrated into a contextually organized spatial representation.

Further to this suggestion that both MEC and LEC process spatial information, recent evidence has suggested that MEC might process global spatial frameworks while local spatial frameworks are represented within LEC (Knierim et al., 2013; Neunuebel et al., 2013). In a double rotation study, Neunuebel et al. (2013) show that LEC neurons were controlled by local spatial features (textured flooring on a circular track) and MEC neurons were controlled by global extra maze cues. Given that place cell activity can be modulated by both local and global landmarks (Knierim and Hamilton, 2011), the MEC and LEC could be providing the hippocampus with varying scales of spatial information.

In the current study, we sought to examine this theory by testing the roles of MEC and LEC in processing global and local spatial frameworks. In the first experiment, spatial frameworks were generated using an arrangement of small objects placed on the testing apparatus (local) or larger objects located around the room (global). Animals were trained and tested on novel food search tasks solvable using either local or global spatial frameworks. Between the training and testing phases, animals received excitotoxic lesions to either the MEC or LEC. It was hypothesized that MEC and LEC lesioned animals would be impaired on the global and local spatial framework task respectively. Here we report that LEC lesioned animals were impaired on the local spatial framework task. MEC lesioned animals were not impaired on either local or global spatial framework task. A second experiment aimed to further investigate the role of entorhinal cortex in local spatial frameworks by examining how animals with LEC and MEC lesions performed on tests of object recognition (OR) of varying levels of complexity. Consistent with our previous findings LEC lesioned animals were specifically impaired on a complex OR task where animals process multiple associations between local features within their environment.

## Materials and Methods

### Subjects

Male lister hooded rats (24 rats, Charles River, UK; 12 rats, Harlan Olac, UK) were housed in pairs (*n* = 36; average weight at start of experiment: 363 g) on a 12-h light/dark cycle. Behavioral testing was conducted 7 days a week during the light phase. Rats were food restricted to no less than 85% of their free-feeding bodyweight to keep them motivated to perform the food search tasks. However, water was made freely available to them in their cages. Rats were handled, maintained and used in accordance with national (Animals [Scientific Procedures] Act, 1986) and international (European Communities Council Directive of 24 November 1986 [86/609/EEC]) legislation as well as local approval from St Andrews Animal Welfare and Ethics Committee. The experiment was run in two replications with 12 animals in replication 1 and 24 animals in replication 2. All animals completed Experiment 1. The 24 animals from replication 2 were also run on Experiment 2.

### Apparatus: Experiment 1

#### Local Task

**Figure 1:**
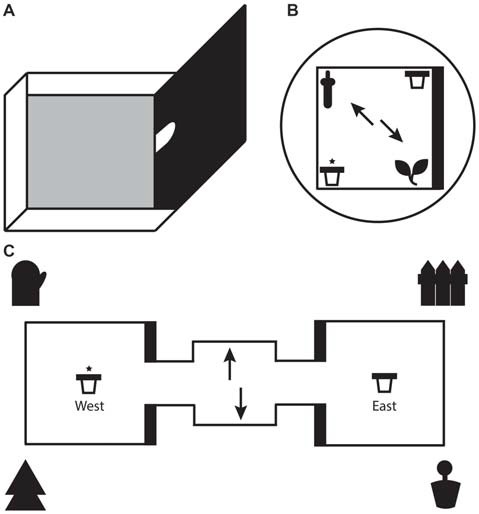
**(A)** Illustration of a single test box with one high wall and three low walls. For the local task, only one test box was used with the arched doorway shut. For the global task, two identical test boxes were connected on either side of a starter box. Animals moved between boxes through the arched doorways in the middle of the high walls. **(B)** Schematic representation of the local task. The test box consisted of two 3D objects (local cues) in opposite corners and two pots of sand (rewarded/unrewarded) in the remaining corners. A black curtain surrounded the test box to eliminate global cues. **(C)** Schematic representation of the global task. One pot of sand each (rewarded/unrewarded) was placed in the center of each test box. Four large global landmarks were placed around the apparatus. **(B,C)** Arrows depict the two potential starting positions on each trial. The reward pot is highlighted with an asterisk. The thick black line represents the high wall of the test box.

For the local spatial framework task (hereon referred to as the local task) one of the test boxes was detached from the apparatus and placed on an individual stand 35 cm above the ground (see Figure 1). Test boxes were alternated on each day of testing to avoid one box containing significantly more odor cues for when the boxes would be re-assembled for the global task. Two objects, a ceramic frog and a plastic bottle, were placed in the box at diagonal corners. The design of the test box meant that one object was located in the corner of a high wall and a low wall and the other object at the intersection of two low walls. The two objects along with their relationship with the geometric layout of the test box represented the local spatial framework. Pots of sand each were placed in the two remaining diagonal corners, with only one of those pots consistently paired with a chocolate weeto (Weetabix Limited, UK) counterbalanced across rats. The objects and pots were fixed to the floor of the box with Dual Lock Velcro (3M, St Paul, MN, USA). To prevent animals from using olfactory cues to solve the task several precautions were taken. Two pots of sand were half filled with weetos and were placed on the floor directly under the center of the test box to mask the smell of the food reward. A thin layer of sand and crushed weetos was spread across the floor of the test boxes and was moved around between each trial to achieve the same result. The test box was surrounded with a circular black curtain to eliminate any global spatial cues. In a similar vein, the test box was rotated by 90° every trial. To ensure that rats did not have the chance to amend an incorrect choice by digging in both bowls, the experimenter had to be on hand to immediately remove the animal at the end of each trial. Therefore, the experimenter stood at different random locations around the test box during every trial to extricate the animal without becoming a consistent global cue. Both objects and pots were wiped down after each trial. A radio was played in the background to remove distracting ambient noise in the lab.

#### Global Task

The apparatus for the global spatial framework task (hereon referred to as the global task) consisted of two identical 50 cm square test boxes connected by a 30 cm square starter box (see Figure 1). All three boxes were detachable from each other. Each test box had 3 low walls (8 cm) and 1 high wall (50 cm). The apparatus itself was placed on a stand 35 cm above the ground and was surrounded by four extra maze cues, one at each corner. The experimenter sat a few feet behind the starter box. The extra maze cues, along with layout of the room and the consistent location of the experimenter, served as the global spatial framework. A pot of sand was attached to the middle of each text box with Dual Lock Velcro (3M, St Paul, MN, USA). One of the pots was consistently baited with a chocolate weeto in the sand (side of rewarded pot counterbalanced across rats). Similar precautions to those taken in the local task were used to mask the smell of the food reward. The starter box and pots were wiped down after every trial and the apparatus itself was rotated by 180° half way through trials. The latter safeguarded against animals using local cues to solve the task by associating the smell of a particular test box with the baited pot.

### Apparatus: Experiment 2

#### Object Recognition Tasks

Two variations of an OR task were run to examine whether OR ability had been disrupted by lesions to the MEC or LEC. In a complex OR task, the apparatus was set up in a similar configuration to the local task but with four different objects in the four corners of the test box (Van Cauter et al., 2013). A standard, simple OR version was run using two objects in a square box with walls of uniform height (for apparatus details, see Wilson et al., 2013a).

### Behavioral Procedure: Experiment 1

Rats were handled in the experimental room for 10 min each on 3 days in the week leading up to the start of the experiment. On another 3 days, two bowls filled one-third with weetos and two-thirds with sand were placed in the rats’ home cage to habituate them to digging for and eating weetos. Habituation to the maze took place for 2 days before the start of each training phase. Animals were habituated to a specific apparatus configuration depending on the task they were going to be trained on. The order of training and testing was counterbalanced across animals (see Supplementary Table S1). Surgery followed 7–10 days after training.

#### Global Task

On each day of testing, rats had to complete eight trials. On each trial, rats were placed in the starter box facing either north or south so that they never started directly facing a passageway leading to one of the test boxes. Rats had to use the extra maze cues to locate the food reward. Local cues including the geometry of the apparatus could not be used to differentiate between the rewarded and unrewarded pots. The reward pot was always in the same location relative to the global spatial framework (either east or west) for each animal. Rats were placed in a holding cage after each trial, which was after they had either eaten the food reward or stopped digging in the incorrect pot. The interval between trials was approximately 60–90 s, which was how long it took the experimenter to reset the apparatus. The starting position, north or south facing, was counterbalanced for each animal. The reward location was counterbalanced for two rats in the same cage and pots were switched between test boxes after every trial, again to ensure rats could not use local cues to solve the task.

#### Local Task

Testing days again consisted of eight trials. In the local task, animals were placed in the test box facing one of the objects and had to learn to dig in the correct pot using local cues such as the objects, wall heights or a combination of the two. The local spatial configuration was maintained such that, for example, the same object was always positioned at the intersection of the low and high wall. The reward was always in the same pot in relation to the local cues. The end of a trial was classified as being when the rat either finished eating the weeto or stopped digging in the incorrect pot. After each trial, animals were removed from the apparatus and placed in a holding cage while the experiment was reset. Inter-trial intervals were similar to those in the global task. On every trial, an animal’s starting position, namely the object he faced, was counterbalanced. Position of reward pots were also counterbalanced between rats within a cage. Within a testing session the pots were swapped to prevent scent marking while the position of the reward relative to the local cues was maintained.

### Behavioral Procedure: Experiment 2

#### Object Recognition Tasks

OR tasks were run with animals from replication 2 (MEC = 10, LEC = 6, Shams = 8). In the complex OR task, animals explored four different objects placed in four corners of the local apparatus. Animals were given six blocks of 4-min explorations. Each block was concluded at the end of 4 min or if the animals explored each object for 15 s each, whichever happened first. Between blocks, animals were kept in a holding cage for 1 min while the objects and the box were cleaned. On the seventh block, three new copies of previously seen objects along with one new object were set up on the apparatus. Animals were given the full 4 min to explore all objects. Novel object identity and the corners in which they were presented were counterbalanced across animals. The standard simple OR task was conducted with the same protocol used in Wilson et al. (2013a). Animals were tested for 4 days with each day consisting of a sample and test phase. In the sample phase, animals explored two identical objects for 3 min. The sample phase concluded at the end of 3 min or when an animal explored both objects for 15 s each, whichever was shorter. Between sample and test phase, animals were placed in a holding cage for 1 min while the box was cleaned. In the test phase, animals explored one new copy of a previously seen object and one new object. Novel object and the location they were presented in were counterbalanced between cage mates and within and across days.

### Surgery

The order in which animals were assigned to lesion or sham groups was counterbalanced to account for learning performance. This was done to prevent a situation where, for example, all MEC lesion animals were those that learned both tasks the quickest. All rats were initially anesthetized in an induction box using isoflurane (Abbot Laboratories, Maidenhead, UK). Anesthesia was maintained with a facemask mounted on the incisor bar (2%–3% isoflurane, 1.2 1/min O_2_) once the animals were placed in the stereotaxic frame (David Kopf, Tujunga, CA, USA). Rimadyl (0.05 ml/rat; 5% w/v carprofen; Pfizer Ltd, Kent, UK) was injected subcutaneously as a pre-surgical analgesic. After shaving the scalp, a midline incision was made and holes were drilled bilaterally at stereotaxic coordinates measured on the skull surface targeting either LEC (AP: −6.5 mm from Bregma; ML: ± 4.5 mm from midline) or MEC (AP: −8.3 mm and −8.8 mm from Bregma; ML: ± 4.8 mm and ± 4.7 mm from midline). Dura was cut using the bent tip of a 30-gauge needle.

For the LEC procedure, a glass pipette (end diameter 30–40 μm) was lowered into the brain at a 10° angle to 6.4 mm below dura. For the lesion group (*n* = 10), 200 nl of ibotenic acid (0.03 M solution made up in sterile 0.1% phosphate buffer; Sigma-Aldrich, UK) was infused by pressure injection and left *in situ* for 5 min after infusion. For the LEC sham controls (*n* = 6), the identical protocol was carried out but only the vehicle solution (sterile phosphate buffer) was injected. For the MEC procedure, a 33-gauge cannula was super glued to the end of a Hamilton syringe and lowered into the brain until it touched the bottom of the skull, after which it was raised up by 0.7 mm. For the lesion group (*n* = 14), two injections of 0.1 μl and 0.2 μl ibotenic acid (0.06 M solution made up in sterile phosphate buffer) respectively were infused. For the MEC sham controls (*n* = 6), the identical protocol was carried out but only the vehicle solution was injected. Once the needle was initially pulled up after hitting the skull, it was left *in situ* for 1 min before injecting 0.02 μl of either ibotenate or sterile phosphate buffer in small quantities across 30 s. After injection, the needle was left *in situ* for 2 min. Animals were allowed 7 days to recover from surgery before behavioral experiments were resumed. On days 1–3 post-surgery, rats were fed wet mash mixed with one drop of Metacam Oral Suspension (Boehringer Ingelheim Vetmedica, Inc.) for every 50 g of bodyweight.

### Perfusion

Rats were humanely euthanized with intraperitoneal injections of 200 mg/ml/kg sodium pentobarbitone (“Dolethal”, Univet, Bucester UK) and transcardially perfused with phosphate buffer saline (PBS; 0.9%). This was followed by a minimum of 300 ml paraformaldehyde solution (4% made up on 0.1% phosphate buffer solution). Brains were then extracted and kept in 20% sucrose solution (made up of 0.1% phosphate buffer) for 7 days.

### Histology and Immunohistochemistry

Brains were immersed in egg yolk within individual plastic wells and placed in a glass bowl containing 4% paraformaldehyde filled one-third of the way up to the wells. The bowl was sealed and left for 5 days to allow the egg to fix onto the outside of the brains. The brains were then cut in 50 μm coronal sections on a freezing microtome with 1:4 being processed for neuronal nuclei (NeuN). For NeuN staining, sections were washed for five rounds of 3 min each in 0.1 M PBS before being placed on a stirrer in blocking solution for 1 h (0.1 M PBS, 20% normal goat serum, 0.1% triton). Sections were then washed again as previously outlined, incubated in anti-NeuN (Chemicon International, Temecula, CA, USA) at a concentration of 1:4000 and left overnight on a stirrer. Sections were washed again before being incubated on a stirrer in vector IgG solution (anti-mouse IgG at 5 μl/ml ADS; Vector Laboratories Ltd, Peterborough, UK) for 1 h. Sections were washed and then incubated on a stirrer in Vectastain ABC complex (Vector Laboratories Ltd, Peterborough, UK; reagents A and B at 10 μl/ml ADS) for an hour. Sections were washed before being immersed in Sigma Fast 3,3′-Diaminobenzidine tablets (DAB; Sigma Chemical Company, St Louis, MO, USA) for approximately 10 min. Sections were removed from DAB when neurons were clearly visible relative to the background staining. Sections were washed one final time, mounted on slides and stored overnight in a formaldehyde bath. Slides were cleared in xylene and then coverslipped with DPX mountant (BDH Laboratory Supplies, Poole, UK).

### Lesion Analysis

Slides were viewed under a light microscope (Leitz Diaplan) at ×4 magnification and lesion extent was judged by the lack of cell bodies or by cells that were shrunken and damaged. ImageJ (v1.50) was used to draw lesion damage onto 8 standardized sections of the MEC (8.28 mm, −8.04 mm, −7.80 mm, −7.68 mm, −7.32 mm, −6.96 mm, −6.60 mm and −6.24 mm from Bregma) and LEC (−7.68 mm, −7.32 mm, −6.96 mm, −6.60 mm, −6.24 mm, −5.88 mm, −5.52 mm and −5.16 mm from Bregma) with reference to Paxinos and Watson (2007) and the online Rat Hippocampus Atlas^1^.

### Behavioral Analysis

#### Days to Criteria

Rats were judged to have completed a local or global task if they found the food reward on at least six out of eight trials (75%) on three consecutive days. Therefore, an animal with a DTC score of 3 on the local task would have secured six or more correct trials on each of the first 3 days of that task and would be eligible to progress onto the global task. This same criterion was used to assess performance both pre-surgery (training phase) and post-surgery (testing phase). Post-surgery, we wanted to assess the performance on both tasks between lesion groups. To account for the difference in task difficulty we used a mean difference score. The difference score was created by subtracting the sham group average DTC score from each lesioned animal’s DTC score to give a difference from the control for each animal. This difference score is averaged to give a mean group difference for each task thus normalizing the score with reference to sham performance.

#### Accuracy and Latency Measures

We looked at the accuracy across both tasks for the LEC, MEC and sham groups on the last 3 days before surgery and the first 3 days after surgery. This was calculated as the total number of successful trials divided by the total number of trials across the 3 days of interest. Response latencies were also measured for both tasks on the first 3 days post-surgery. This was achieved by recording how long (in seconds) it took animals to dig in a pot from the moment they were placed on the apparatus.

#### Re-Exploration Score

Animals were judged to be actively exploring objects on both OR tasks when their nose was near the objects. A re-exploration score was used to measure performance on the complex OR task. This was calculated as the time spent exploring the novel object minus the average time spent exploring across the other three familiar objects during block seven (Van Cauter et al., 2013). Exploration times were scored offline by a trained experimenter who was blind to the lesion groups.

#### Discrimination Index

In the simple OR task, exploration times were converted into discrimination indices (discrimination index = (time at novel object − time at familiar object)/(time at novel object + time at familiar object)) to assess OR between a novel and a familiar object. This is equivalent to the D2 measure used by Dix and Aggleton (1999).

### Statistical Analysis

A paired samples *t*-test was conducted using DTC scores from the local and global tasks (pre-surgery) to examine the difficulty of tasks using different spatial frameworks. A 2 × 2 mixed ANOVA, with lesion (MEC vs. LEC) as between subjects factor and task (local vs. global) as within subjects factor, was performed on the mean difference scores (post-surgery) to determine the effect of MEC and LEC lesions on task performance. *Post hoc* analyses used paired samples *t*-tests and independent samples *t*-tests to examine performance within and across groups. To analyze the impact of lesions on task accuracy, a 3 × 2 × 2 mixed ANOVA was conducted for the last 3 days pre-surgery and the first 3 days post-surgery, with group as a between subjects factor (sham vs. MEC vs. LEC) and time (pre-surgery vs. post-surgery) and task (local vs. global) as within subjects factors. *Post hoc* paired samples *t*-tests were used to investigate the effect of accuracy on both tasks prior to and post-surgery. A 3 × 3 × 2 mixed ANOVA was conducted on response latency during the first 3 days of the test phase, with group as a between subjects factor (sham vs. MEC vs. LEC) and day (D1 vs. D2 vs. D3) and task (local vs. global) as within subjects factors, to assess the impact of lesions on time taken by animals to make a digging choice. One-way ANOVAs were used to assess the effect of lesion group on object exploration in the simple and complex recognition tasks. One sample *t*-tests were conducted for each group during OR tasks to determine whether animals were exploring the novel object above chance.

## Results

### Histology

**Figure 2:**
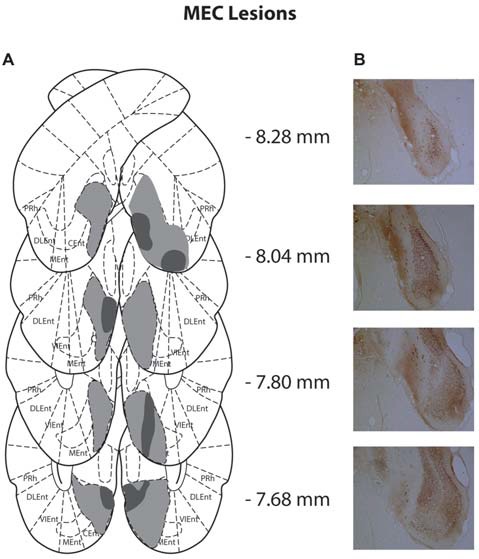
**(A)** Schematic representation of medial entorhinal cortex (MEC) lesions for rats with the greatest (light gray) and least (dark gray) damage. Representations of coronal sections have been adapted from Paxinos and Watson (2007) at −8.28 mm, −8.04 mm, −7.80 mm and −7.68 mm, Bregma, from top to bottom, respectively. **(B)** Photographs of coronal sections of the largest MEC lesions from −8.28 mm, −8.04 mm, −7.80 mm and −7.68 mm Bregma (top to bottom).

**Figure 3:**
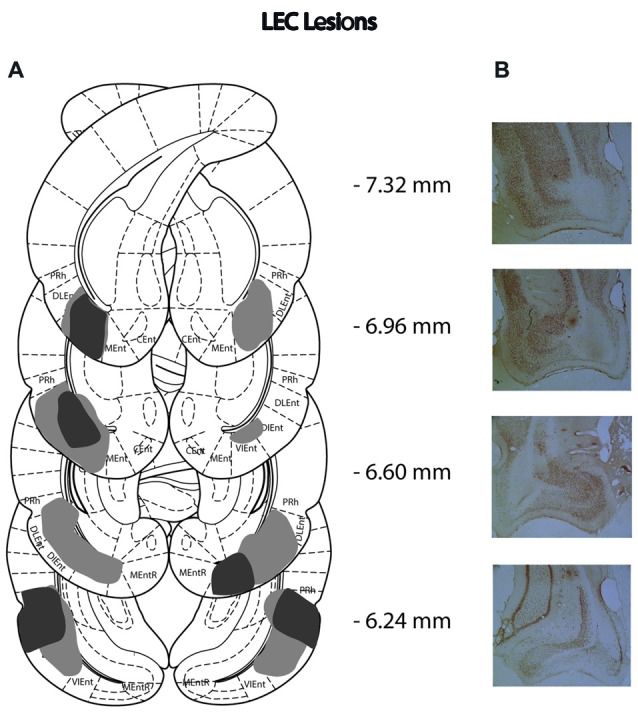
**(A)** Schematic representation of lateral entorhinal cortex (LEC) lesions for rats with the greatest (light gray) and least (dark gray) damage. Representations of coronal sections have been adapted from Paxinos and Watson (2007) at −7.32 mm, −6.96 mm, −6.60 mm and −6.24 mm Bregma, from top to bottom, respectively. **(B)** Photographs of coronal sections of the largest LEC lesions from −7.32 mm, −6.96 mm, −6.60 mm and −6.24 mm Bregma (top to bottom).

Of the 14 rats that received MEC lesions, one animal was excluded (replication 1) for lack of visible damage and another had evidence of a unilateral lesion (replication 2). The largest and smallest lesions from the remaining 13 animals are presented in Figure 2. Five animals had lesion damage that extended to the LEC but this was estimated to be <5% of the total area. MEC lesions also marginally encroached upon parasubiculum, postrhinal cortex and subiculum. Among the LEC lesioned rats, two were excluded (replication 2), one for lack of significant lesions (<5%) and another for extensive damage to the MEC due to surgeon error resulting in excess toxin being injected. For the remaining eight rats the largest and smallest lesions are presented in Figure 3. For one animal, lesion damage extended to the MEC but was estimated to be <5% of the total area. In most cases, minor lesion damage was recorded in the perirhinal cortex and/or hippocampus (subiculum, CA1, CA2). Animals with sham lesions did not show signs of any lesion damage or cell death.

### Behavioral Analysis: Experiment 1

#### Local Task is More Difficult than Global Task

**Figure 4:**
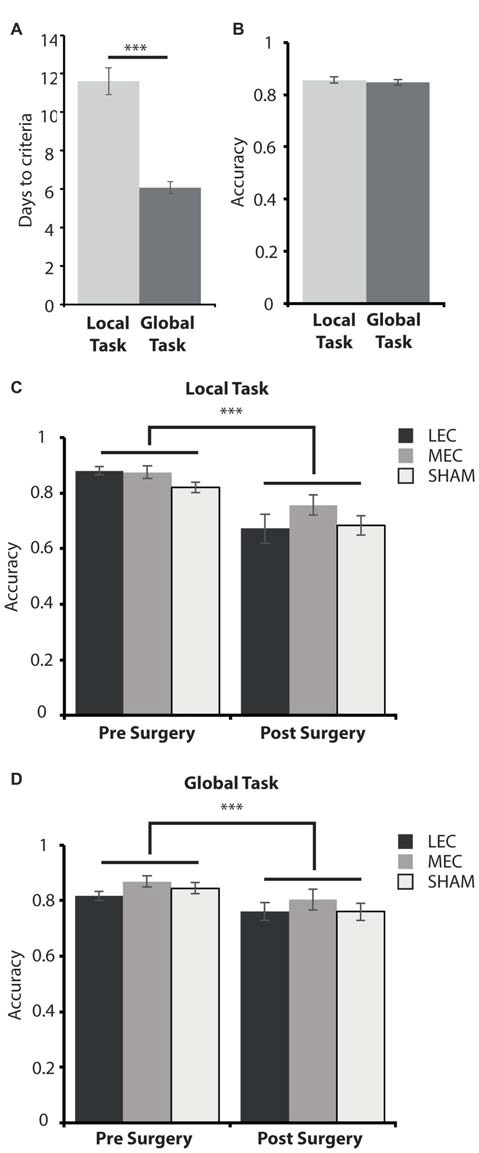
**(A)** Average days to criteria score on the local and global task during the training phase, pre-surgery. **(B)** Average accuracy measure for the last 3 days pre-surgery on the local and global tasks. **(C)** Average accuracy measure for the last 3 days pre-surgery (left) and the first 3 days post-surgery (right) on the local task for LEC, MEC and sham groups. **(D)** Average accuracy measure for the last 3 days pre-surgery (left) and the first 3 days post-surgery (right) on the global task for LEC, MEC and sham groups. ***P < 0.001.

We first examined the difficulty of each task by comparing the number of days it took animals to learn them and the average performance on the criterion days. Figure 4A shows that during the training phase, animals took significantly longer to reach criteria on the local task (paired samples *t*-test: *t*_(32)_ = 6.141, *P* < 0.0001). This demonstrates that the local spatial framework task is harder to learn than the task requiring a global spatial framework.

#### All Animals Are Less Accurate on Both Tasks Immediately after Surgery

As animals took longer to learn one task over the other, we wanted to assess how accurate they were on both tasks when they met criteria before surgery and whether accuracy was impacted for lesioned rats relative to shams immediately after surgery. A 3 (Group) × 2 (Task) × 2 (Time; pre-surgery vs. post-surgery) mixed ANOVA revealed a significant main effect of time (*F*_(1,30)_ = 61.086, *P* < 0.001, partial *η*^2^ = 0.671) and a significant time × task (*F*_(1,30)_ = 10.127, *P* < 0.01, partial *η*^2^ = 0.252) interaction. There was no significant main effect of lesion group (*F*_(2,30)_ = 2.556, *P* = 0.094, partial *η*^2^ = 0.146) or task (*F*_(1,30)_ = 1.908, *P* = 0.177, partial *η*^2^ = 0.060) as well as no significant group × task (*F*_(2,30)_ = 0.321, *P* = 0.728, partial *η*^2^ = 0.021), group × time (*F*_(2,30)_ = 0.670, *P* = 0.519, partial *η*^2^ = 0.043) or group × task × time (*F*_(2,30)_ = 1.261, *P* = 0.298, partial *η*^2^ = 0.078) interaction effects. *Post hoc* tests showed that there was no significant difference in accuracy performance between the local and global tasks on the last 3 days pre-surgery (*t*_(32)_ = 0.526, *P* = 0.603; Figure 4B). This suggests that animals learnt both tasks to a similar level despite the local task taking longer to learn than the global task pre-surgery. Animals were significantly less accurate on the first 3 days post-surgery compared to the last 3 days pre-surgery on both the local (*t*_(32)_ = 6.614, *P* < 0.001) and global (*t*_(32)_ = 4.462, *P* < 0.001, Figures 4C,D) tasks demonstrating some forgetting of the tasks had occurred although this was the same for all three groups.

#### LEC Lesions Impair Performance on a Local Spatial Framework Task

**Figure 5:**
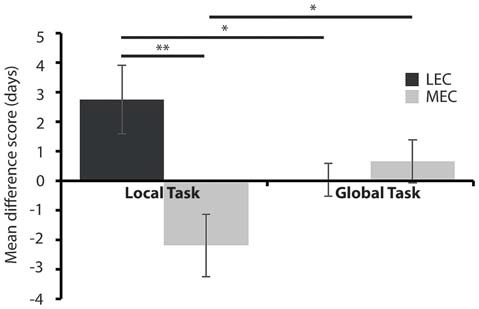
**Average days to criteria mean difference score for MEC and LEC lesioned animals on the local (left) and global (right) task during the test phase, post-surgery.** *P < 0.05, **P < 0.01.

None of the groups maintained criterion level performance from pre-surgery in the immediate post-surgery testing and so we next sought to examine how quickly the animals could relearn the tasks and whether performance was different in rats with LEC and MEC lesions relative to controls. Given the difference in difficulty levels of the two tasks we used a mean difference score. Figure 5 shows that animals with LEC lesions took longer to learn the local task than animals with lesions of MEC while they learned the global task in an equivalent time period. This was confirmed with a 2 × 2 mixed ANOVA on the mean difference scores during the test phase, post-surgery. The analysis revealed a significant lesion × task interaction effect (*F*_(1,19)_ = 11.540, *P* < 0.005, partial *η*^2^ = 0.378) but no main effect of lesion (*F*_(1,19)_ = 3.852, *P* = 0.064, partial *η*^2^ = 0.169) or task (*F*_(1,19)_ = 0.007, *P* = 0.934, partial *η*^2^ = 0.0003). *Post hoc* analysis showed that LEC lesioned rats were significantly slower at learning the local task than the global task (*t*_(7)_ = 2.358, *P* = 0.05) while MEC lesioned rats were significantly faster at learning the local task than the global task (*t*_(12)_ = −2.667, *P* < 0.05). Together, these results indicate that LEC lesioned animals were significantly impaired on the local task relative to the global task while performance of MEC lesioned animals on the local task was facilitated relative to the global task. Further *post hoc* analysis showed that LEC lesioned rats were significantly slower at learning the local task than MEC lesioned rats (*t*_(19)_ = 3.017, *P* < 0.01) but there was no difference in the amount of time taken to learn the global task between lesion groups (*t*_(19)_ = −0.604, *P* = 0.553). These findings were confirmed in analysis of the uncorrected DTC scores where animals with LEC lesions were impaired relative to shams on the local task (*t*_(19)_ = 1.870, *P* = 0.043) while all other comparisons of lesion groups with sham groups showed no significant differences in DTC.

#### Neither MEC nor LEC Lesions Impair Response Latencies

Response latencies in rewarded tasks can provide an interesting insight into decision making and motivation with faster responses potentially suggesting that animals are either more certain of their response or more motivated to find a food reward. To examine these possibilities, we next looked at response latencies of the three groups in the different tasks. There was no difference across groups for response latency during the first 3 days of the test phase, post-surgery. This was confirmed using a 3 (Group) × 3 (Day) × 2 (Task) mixed ANOVA. There was no significant interaction effect of Group × Day × Task (*F*_(4,36)_ = 0.815, *P* = 0.496, partial *η*^2^ = 0.083), Group × Task (*F*_(2,18)_ = 0.246, *P* = 0.784, partial *η*^2^ = 0.027), Group × Day (*F*_(4,36)_ = 0.732, *P* = 0.576, partial *η*^2^ = 0.075) or Task × Day (*F*_(2,36)_ = 3.515, *P* = 0.057, partial *η*^2^ = 0.163). There was also no main effect of group (*F*_(2,18)_ = 0.319, *P* = 0.731, partial *η*^2^ = 0.034). However, there was a main effect of task (*F*_(1,18)_ = 10.132, *P* = 0.005, partial *η*^2^ = 0.360) and day (*F*_(2,36)_ = 7.540, *P* = 0.007, partial *η*^2^ = 0.295). This demonstrates that while neither lesion affected rats’ latency to carry out the tasks the local task was faster to complete than the global and that the rats got faster over days. Neither of these affects is surprising. The global task involves running a longer distance and so we would expect slower latency to dig. The change of latency across days suggests that the rats are learning the task. Critically the lack of effect of lesion suggests that the animals with both lesions are equally motivated to complete the task.

### Behavioral Analysis: Experiment 2

#### LEC Lesions Do Not Impair Simple Object Recognition but Do Impair Recognition of Complex Object Configurations

**Figure 6:**
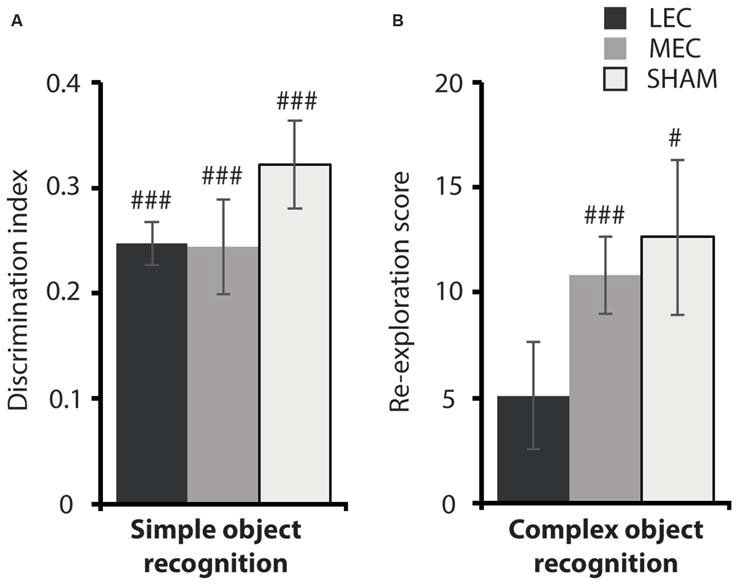
**(A)** Average discrimination indices for the MEC, LEC and sham groups on the simple object recognition (OR) task. **(B)** Average re-exploration scores for the MEC, LEC and sham groups on the complex OR task. ^#^*P* < 0.05, ^###^*P* < 0.001, *t*-test vs. 0 (simple OR), *t*-test vs. 0 (complex OR).

Given that LEC lesioned animals were impaired on the local task, we wanted to ascertain if this deficit was a result of compromised OR. Figure 6 depicts performance on the two OR tasks. As previously reported, there was no difference in object discrimination across groups in the simple OR task (Figure 6: *F*_(2,18)_ = 0.941, *P* = 0.409, partial *η*^2^ = 0.095). Further, all animals explored the novel object above chance (LEC: *t*_(3)_ = −0.26, *P* < 0.001; MEC: *t*_(9)_ = −0.26, *P* < 0.001; Sham: *t*_(6)_ = −0.18, *P* < 0.001). For the complex OR task, one animal from the LEC group was removed from testing because of a swollen foot. Again, we did not see a difference in novel object re-exploration across lesion groups (*F*_(2,17)_ = 1.036, *P* = 0.375, partial *η*^2^ = 0.103). However, LEC lesioned animals performed at chance level on this task (*t*_(2)_ = 5.11, *P* = 0.076) compared to animals in the MEC (*t*_(9)_ = 10.83, *P* < 0.001) and Sham (*t*_(7)_ = 12.62, *P* < 0.05) groups who both performed above chance levels. The results from both OR tasks indicate that LEC lesions impaired recognition of a complex configuration of objects but not a simple discrimination between two objects.

## Discussion

In the current study, we sought to test the hypothesis that both entorhinal inputs to hippocampus are involved in processing spatial information but at different scales with MEC and LEC processing global and local spatial information respectively. Our studies show that lesions of LEC impaired rats’ ability to solve a task using a local spatial framework. Consistent with our previous work LEC lesions did not disrupt the ability of animals to recognize individual objects in a standard test of OR but interestingly did impair their ability to recognize a novel object within a complex local environment. LEC lesioned rats performed as well as control animals on the global task demonstrating a specific role for LEC in processing local spatial features. MEC lesions, however, did not impair performance on either spatial framework task or on either test of OR. Indeed, the only effect of MEC lesions in the current data set was a facilitation of performance on the local task. Our data provide support for the theory that LEC is involved in processing local spatial features but surprisingly does not clearly support the suggestion that MEC is involved in processing global features of the environment.

### Spatial Learning Using Local vs. Global Frameworks

One interesting aspect of the data concerns task difficulty and gives us an interesting insight into how animals preferentially use cues to navigate. Here, we show that animals took longer to learn the local task irrespective of whether they were trained before or after the global task. This finding is in line with previous studies demonstrating that local cues are less salient when defining a spatial environment (Gothard et al., 1996; Benhamou and Poucet, 1998; Save and Poucet, 2000). One possible explanation for this has been put forward by Hamilton et al. (2007, 2009). The authors propose that global and local cues provide direction and place information respectively in a sequential order. For example, animals may initially rely on global cues to orient the direction of their trajectory within an apparatus and then use local cues to pinpoint specific locations. Evidence for sequential encoding of space has also been shown in humans (Hamilton et al., 2009). The same authors tracked eye fixations in a virtual Morris water task and found that participants initially focused on external, room cues at the start of the trials before switching attention to the apparatus. This is also consistent with self-reported evidence from interviews conducted as part of a cognitive mapping exercise across various American cities (Lynch, 1960). When asked to re-count their typical journeys around the city, new residents included a greater number of global landmarks than local cues in their recollection. This effect was reversed when more experienced residents were interviewed. In our experiment, slower learning performance on the local task could be explained as the animals’ inability to initially encode any global cues that would be useful in orienting themselves to a new environment. This suggests that our task involved the use of two different spatial frameworks and the complexity of these tasks is in line with previous research using similar paradigms.

### Role of LEC in Processing Spatial Frameworks

Rats with LEC lesions were impaired on a local spatial framework task. Our current findings, along with those from past research, suggest that the LEC is recruited to provide spatial information that is object-orientated, potentially multimodal and associative in nature (Deshmukh and Knierim, 2011; Deshmukh et al., 2012; Tsao et al., 2013; Van Cauter et al., 2013; Wilson et al., 2013b). Previous work from our lab has shown that LEC lesions impair rats’ ability to associate any combination of objects, places and the contexts in which they are experienced (Wilson et al., 2013b). These data suggest that LEC is necessary for integrating features of an event including local spatial information. Consistent with this Chao et al. (2016) reported impairments in integrating features of an event, including place, object and time information, in animals with contralateral lesions of medial prefrontal cortex (mPFC) and LEC. Forming associations between objects and the locations, contexts and times in which they have been experienced is a crucial part of developing and maintaining a spatial framework. This ability is also crucial for remembering specific episodes from our lives implicating LEC not only in spatial processing but also in episodic memory.

Is the role of the LEC then best characterized as a local spatial deficit or an associative deficit? Our results show that LEC lesioned animals are not impaired on standard tasks of OR involving discrimination between two objects. Our previous studies have also shown that rats with lesions of LEC are capable of recognizing when a familiar object has moved locations when there are only two identical objects in the task. This suggests that LEC lesioned rats are capable of processing individual aspects of their experience and that their deficit becomes manifest when they are required to associate these things together. Given that local spatial frameworks involve an association of features this explains why LEC lesioned rats are impaired at performing a task requiring integration of local spatial cues. Our finding that LEC lesioned animals are impaired at discriminating complex object configurations is consistent with recent work by Save and colleagues (Van Cauter et al., 2013; Rodo et al., 2017). The authors show that animals with LEC lesions can discriminate a novel object in configurations of three but not four different objects. They interpret their findings in terms of LEC being needed in situations where complexity is increased. Our data could also be interpreted in these terms. When rats need to associate multiple aspects of their environment this increases the complexity and causes deficits in LEC lesioned animals.

If the LEC has a general role in associative memory though why are animals with LEC lesions not impaired on the global task that also involves using a spatial framework? This pattern of results whereby LEC is needed to integrate stimuli that are close to the animal in the local environment such as objects could be the result of the multimodal sensory information it receives. The hypothesis that LEC is the hub for integrating multisensory information into memory is entirely consistent with anatomical studies of the network. In a recent network analysis of over 16,000 articles examining the histologically defined connections between cortical regions in the rat, Bota et al. (2015) identified the LEC as one of three hubs within the cortical association network, along with the ectorhinal and perirhinal areas, stating that “The lateral entorhinal area forms the richest set of association connections of any cerebral cortical region in rat”. The LEC receives extensive connections from areas processing olfactory information, most notably the piriform cortex, as well as receiving strong connections from the other hubs of the cortical association network. Similar findings have been reported in mice (Zingg et al., 2014). Multimodal information including olfactory and tactile stimuli is usually only available when examining objects that are within our immediate proximity. It is possible, therefore, that the evidence showing a specific role for LEC in local spatial frameworks is due to the types of sensory input it is receiving.

### Role of MEC in Processing Spatial Frameworks

Contrary to our hypothesis, MEC lesioned animals were not impaired on the global spatial framework task. This suggests that MEC is not needed to process global allocentric spatial information. The only significant effect of MEC lesions was an apparent facilitation of the local task. While it might be interesting to posit that removing the MEC removes the system that usually processes global cues, thus allowing animals to concentrate on local cues, this is not supported by the lack of deficit on the global task. This finding is not consistent with recording studies that have reported spatially modulated grid cells, head direction cells, conjunctive cells, border cells and speed cells in MEC (Hafting et al., 2005; Sargolini et al., 2006; Solstad et al., 2008; Kropff et al., 2015). One possible explanation for a lack of spatial deficits is the relative lesion size in the MEC group. It could be the case that the portion of MEC left intact would be sufficient to support spatial memory. However, it should be noted that our lesions did extend to the dorsolateral MEC, an area with a high prevalence of grid cells (Hafting et al., 2005) and one previously shown to be important for recall of spatial reference memory based on a global spatial framework (Parron et al., 2004; Steffenach et al., 2005). It should also be noted that studies reporting greater MEC lesion coverage have described either a lack of spatial deficits (Bannerman et al., 2001; Burwell et al., 2004) or deficits that are classified as mild (Van Cauter et al., 2013).

An alternate explanation for lack of spatial impairments in MEC lesioned rats could be the task requirements. It is possible that the global task required animals to use memory for a well learned spatial reference with minimal reliance on navigation mechanisms such as grid cells and border cells. Such a task could be solved using systems located outside of MEC. Head direction cells found in multiple brain regions (Taube, 2007) allow an animal to orient itself in the environment while place cells in the hippocampus encode landmarks. As previously mentioned, it has been shown that animals and humans initially use global cues to provide directional orientation when navigating (Hamilton et al., 2009). As such, animals in our experiment could orient themselves to the correct test box using head direction cells guided by one of the global cues. Indeed it has been demonstrated that animals with MEC lesions have normal head direction cells in the anterodorsal thalamus that continue to be controlled by global landmarks (Clark and Taube, 2011). It is possible, therefore, for animals to solve our global task by orienting themselves to individual landmarks with the help of head direction cells beyond the MEC. Another region that could be contributing towards generating a global framework even in the absence of MEC is the hippocampus. Although reciprocal connections link hippocampus with the MEC, place cells appear to exert greater influence on MEC, than the other way around. Muscimol inactivation of the hippocampus results in an alteration of grid cell firing fields (Bonnevie et al., 2013) while lesioning the MEC does not completely disrupt place cell firing (Brun et al., 2002, 2008; Van Cauter et al., 2008; Hales et al., 2014). Further, from a developmental perspective, it has been shown that place cell expression precedes that of grid cells in developing rat pups (Langston et al., 2010; Wills et al., 2010). These studies demonstrate that a combination of head direction from outside of MEC combined with landmark information from hippocampal place cells could allow rats in this task to solve the task. An interesting question for future studies is how the spatial representations within MEC are used to support specific navigation and spatial behaviors.

## Author Contributions

MVK and JAA designed the studies, ran the experiments, analyzed data and wrote the article.

## Conflict of Interest Statement

The authors declare that the research was conducted in the absence of any commercial or financial relationships that could be construed as a potential conflict of interest.

## Footnotes

^1^www.rbwb.org
