# Supplementary material for: Lateral Entorhinal Cortex Lesions Impair Local Spatial Frameworks
Source: Front Syst Neurosci. 2017 May 17;11:30. doi: 10.3389/fnsys.2017.00030 (PMC5434111; doi:10.3389/fnsys.2017.00030)
Supplement: Supplementary file 1 [file Table_1.docx]

Supplementary Material

Lateral entorhinal cortex lesions impair local spatial frameworks

Maneesh Varghese Kuruvilla, James Alexander Ainge^*^

*** Correspondence:** Dr. James A. Ainge: jaa7@st-andrews.ac.uk

# Supplementary Figures and Tables

Table 1: Counterbalanced combinations of training (pre-surgery) and testing (post-surgery) order on the local and global tasks

| **Training order pre-surgery** | | | | **Testing order post-surgery** | |
| --- | --- | --- | --- | --- | --- |
| **Habituation** | Task | Habituation | Task | Task | Task |
| Local | Local | Global | Global | Local | Global |
| Local | Local | Global | Global | Global | Local |
| Global | Global | Local | Local | Global | Local |
| Global | Global | Local | Local | Local | Global |
